# Unraveling the mitochondrial genome of *Quercus litseoides*: a step towards conservation of an endangered species

**DOI:** 10.3389/fpls.2025.1620373

**Published:** 2025-08-19

**Authors:** Ruo-Han Shen, Yu Li, Liang-Hai Yang, Si-Si Zheng, Xu Yan, Gregor Kozlowski, Xi-Ling Dai, Yi-Gang Song

**Affiliations:** ^1^ College of Life Sciences, Shanghai Normal University, Shanghai, China; ^2^ Eastern China Conservation Centre for Wild Endangered Plant Resources, Shanghai Chenshan Botanical Garden, Shanghai, China; ^3^ Department of Biology and Botanic Garden, University of Fribourg, Fribourg, Switzerland; ^4^ Natural History Museum Fribourg, Fribourg, Switzerland

**Keywords:** *Cyclobalanopsis*, mitochondrial genome, Fagaceae, repeated sequence, phylogenetic relationship

## Abstract

**Introduction:**

Compared to the large number of chloroplast genome resources in *Quercus*, only six mitogenomes (belonging to three sections) have been reported. To date, no mitogenome has been reported for *Quercus* section *Cyclobalanopsis. Quercus litseoides*, a representative species whose chloroplast genome has been characterized, is an endangered tree endemic to the montane cloud forests of southern China.

**Methods:**

In this study, we assembled and annotated the mitogenome of section *Cyclobalanopsis* (*Q. litseoides*) for the first time using the HiFi reads. We examined repeat sequences, codon usage bias, RNA editing events, and chloroplast to mitochondrion DNA transfer events, and performed collinearity analysis and phylogenetic analysis with other Fagaceae species.

**Results:**

The mitogenome of *Q. litseoides* revealed a multipartite structure composed of three continuous segments with 516,686 bp in length. The genome encoded 38 protein-coding genes, 23 transfer RNA genes, and three ribosomal RNA genes. Repeat analysis uncovered diverse simple sequence repeats and interspersed sequences, and codon usage showed clear biases. Nonsynonymous sites of RNA editing showed 12 different effects on amino acids. Notably, a small amount (1.20%) of DNA sequences occurred gene transfer events between organelles in *Q. litseoides*. Comparative synteny analysis revealed substantial structural variation among oak mitogenomes. *Quercus litseoides* was closely related to *Q. cerris* in both the mitochondrial and chloroplast trees.

**Discussion:**

This work fills a critical gap in mitochondrial genomic resources for *Quercus* section *Cyclobalanopsis*, and provides new insights into the structural diversity and evolutionary dynamics. It also establishes a valuable genomic foundation for phylogenetic reconstruction, adaptive evolution research, and the conservation of endangered *Quercus* species.

## 1 Introduction

*Quercus*, the largest genus in Fagaceae family, is considered ecologically successful, partly due to the evolution of functional genes involved in both physical and chemical plant defense (Wang et al., 2025a). *Quercus* is divided into two subgenera: *Quercus* and *Cerris*. Subgenus *Quercus* includes sections *Lobatae*, *Quercus*, *Protobalanus*, *Ponticae*, and *Virentes*, while subgenus *Cerris* comprises sections *Cyclobalanopsis*, *Cerris*, and *Ilex* (Hipp et al., 2020). Among these, section *Cyclobalanopsis* (cycle-cup oaks) is a dominant element of East Asian subtropical evergreen broad-leaved forests (EBLFs), a biome known for its high biodiversity and specificity (Deng et al., 2018). The formation and evolutionary history of EBLFs are closely linked to major geological and climatic events, such as the uplift of the Qinghai-Tibet Plateau and the development of the East Asian monsoon system (Meng et al., 2025). Enhanced precipitation since the early Miocene promoted the rapid radiation of section *Cyclobalanopsis* in tropical and subtropical evergreen broad-leaved forests of East and Southeast Asia (Jin et al., 2024).

Among the diverse taxa of section *Cyclobalanopsis*, certain species with restricted distributions and specialized ecological niches warrant special attention due to their vulnerability to environmental change (Foster, 2001; Song et al., 2019). *Quercus litseoides* Dunn, an evergreen shrub endemic to southern China, is a representative species among them. Morphologically, its leaves are obovate-lanceolate or narrowly elliptic; the nuts are ellipsoid with sparse pubescence at the apex, and the bowl-shaped cupules cover approximately one-third of the nut (Huang et al., 1999). It is currently known from only six scattered populations in montane cloud forests (700–1000 m a.s.l.) of southern Guangdong and Hong Kong, China (CFH, 2022; Huang et al., 1999). The survival of these populations is severely threatened by these biological and ecological vulnerabilities, combined with habitat destruction, soil erosion, and climate change (Carrero et al., 2020). According to the IUCN Red List of Threatened Species (2020), *Q. litseoides* is assessed as Vulnerable (VU) under criterion B2ab (iii) (Carrero and Strijk, 2020; Carrero et al., 2020). Due to its limited dispersal capacity and dependence on specific ecological environment, *Q. litseoides* is highly sensitive to environmental fluctuation. Consequently, effective conservation measures are urgently needed.

Chloroplasts and mitochondria are two semiautonomous organelles with independent genomes originating from ancient endosymbiotic events, and are typically uniparentally inherited in plants (Cheng et al., 2021; Gould et al., 2008; Margulis, 1970). These two organelles coexist exclusively in higher plants and show significant differences in their structural composition, functional characteristics, and evolutionary dynamics (Wang et al., 2024a). In terms of structural composition, nearly all chloroplast genomes in plants possess a highly conserved circular quadripartite structure, generally ranging from 120 to 160 kb in length (Palmer, 1985; Shaw et al., 2007). In contrast, the plant mitogenomes have experienced the most fluctuating variations in genome size (ranging from 66 kb to 19 Mb, with up to 200-fold variation) (Huang et al., 2025b; Oda et al., 1992), molecular structure (comprising various forms such as circular, linear, and branched molecules) (Guo et al., 2024; Kong et al., 2025; Yang et al., 2023), and sequence composition (with relatively conserved coding regions but highly variable intergenic regions) (Ma et al., 2022; Sheng, 2025; Wang et al., 2025b). In terms of functional characteristics, chloroplasts play a crucial role in photosynthesis and carbon fixation (Bobik and Burch-Smith, 2015), while mitochondria are essential for respiration and metabolism by converting biomass energy into chemical energy through phosphorylation (Klingenberg, 2008; Kroemer and Reed, 2000). In addition, plant mitochondria also play a key role in the development of cytoplasmic male sterility (CMS), a trait caused by mutation or rearrangement of the mitogenome and widely utilized in hybrid breeding systems (Chen et al., 2017, Chen et al., 2021; Han et al., 2024). Finally, in terms of evolutionary dynamics, chloroplast genomes generally exhibit a low genetic recombination rate and a moderate molecular evolution rate, and are widely used in phylogenetic studies (Li et al., 2025a; Liu et al., 2023; Wolfe et al., 1987). In contrast, mitogenomes evolve at a much slower nucleotide substitution rate, yet display unusually frequent gene rearrangements and the formation of structural isoforms (Bi et al., 2023; Christensen, 2013; Wang et al., 2024a). This evolutionary pattern is considered to be one of the unique characteristics of plant mitogenomes, and its evolutionary driving force is the result of the synergistic action of multiple mechanisms (Wang et al., 2024c).

These unique features have stimulated growing interest in plant mitogenomes, particularly for investigating structural diversity, elucidating phylogenetic relationships, and exploring RNA editing and gene transfer events (Dong et al., 2023; Hu et al., 2023; Kong et al., 2025; Xiao et al., 2025). However, the complexity of their genomic architecture continues to pose challenges and has led to their slower progress compared to chloroplast genome research (Wang et al., 2024a). Despite the rapid accumulation of over ten thousand chloroplast genomes in public databases, the number of fully assembled and annotated plant mitogenomes lags far behind, with only a few hundred currently available (Wang et al., 2024a). This discrepancy is also evident in *Quercus*, where chloroplast genomes have been assembled for 124 species, whereas mitogenome sequences have been published for only seven species (*Quercus acutissima*, *Q. cerris*, *Q. chenii*, *Q. ilex*, *Q. petraea*, *Q. robur*, and *Q. variabilis*) to date. This disparity highlights the urgent need for more comprehensive mitogenomics research. These available mitogenomes have revealed considerable diversity in genome architecture, repeat content, and gene retention patterns (Bi et al., 2019; Liu et al., 2022). However, section *Cyclobalanopsis*, despite its high ecological significance in East and Southeast Asia, remains entirely unexplored in terms of mitogenome research. This gap has hindered our understanding of its genomic architecture, evolutionary dynamics, and phylogenetic implications. Therefore, assembling and analyzing mitochondrial genomes from section *Cyclobalanopsis* is crucial.

Although the chloroplast genome of *Q. litseoides* and the phylogenetic relationship of *Quercus* section *Cyclobalanopsis* have been studied recently (Li et al., 2022, Li et al., 2024), no mitogenome has been reported for *Q. litseoides*. To address this gap, we report and characterize the complete mitogenome of *Q. litseoides*, representing the first mitogenome of *Quercus* section *Cyclobalanopsis*. The primary objective is to assemble and annotate the mitogenome of *Q. litseoides* using high-throughput sequencing data. Based on this mitogenome, we aim to: (1) analyze the structural features and repeat sequences; (2) assess codon usage bias in protein-coding genes; (3) identify potential inter-organellar gene transfer events; (4) predict RNA editing sites; and (5) examine syntenic relationships with other published Fagaceae mitogenomes. Additionally, we reconstruct a mitogenome-based phylogenetic tree to explore the evolutionary placement of *Q. litseoides* within *Quercus*. This study provides important theoretical support and genomic data for the conservation and taxonomic research of the endangered species *Q. litseoides*. Moreover, this work not only provides the first mitogenomic resource for section *Cyclobalanopsis*, but also expands our understanding of organellar genome evolution in *Quercus*.

## 2 Material and methods

### 2.1 Plant materials and sequencing

Fresh blades of *Q. litseoides* were collected from Wutong Mountain, Shenzhen (113°17′E, 22°23′N; Alt. 943.7 m a.s.l), immediately frozen in liquid nitrogen, and stored at -80°C until DNA extraction. Total genomic DNA was extracted using the improved CTAB method (Porebski et al., 1997). The size and integrity of the extracted DNA were evaluated by electrophoresis on a 0.75% agarose gel, which enables assessment of DNA fragmentation and degradation. DNA purity and concentration were assessed using a NanoDrop One spectrophotometer (Thermo Fisher Scientific, USA) and a Qubit 3.0 fluorometer (Life Technologies, Carlsbad, CA, USA), respectively.

Prior to conducting Single Molecule Real Time (SMRT) sequencing, high-quality genomic DNA underwent stringent quality control. The genomic DNA was fragmented into large fragments, and then damage repair, adapter ligation, and fragment selection were performed to construct a PCR-free SMRT bell library. After size selection and quantification, the SMRT bell library was sequenced on the PacBio Revio platform. Following quality control and filtering using the SMRT Link v12.0 software, the sequencing yielded 20.16 Gb of high-throughput third-generation data.

### 2.2 Genome assembly and annotation

The third-generation HiFi data was assembled using PMAT software (https://github.com/bichangwei/PMAT.git) (Bi et al., 2024), yielding a draft mitogenome assembly in Graphical Fragment Assembly (GFA) format. The GFA file was visualized using Bandage software (Wick et al., 2015) to examine the overall genome structure and detect complex connections mediated by repeat sequences. The final mitogenome was obtained by manually identifying and resolving circular molecules through tracing valid paths in the graph. To assess the accuracy and completeness of the mitogenome assembly, we realigned the PacBio HiFi long reads to the assembled mitogenome and calculated the coverage depth for each of the three molecular forms. The coverage plots were subsequently visualized using R. The assembly was annotated using the online annotation tool PMGA (http://www.1kmpg.cn/pmga/) (Li et al., 2025b). The annotation errors were carefully checked and manually corrected using Geneious R9.0.2 software (Kearse et al., 2012). Finally, the mitogenome map was drawn using the Plant Mitochondrial Genomes Map (PMGmap) (http://www.1kmpg.cn/pmgmap) (Zhang et al., 2024).

### 2.3 Repeated sequences analyses

Simple sequence repeats (SSRs) were identified utilizing the MISA web service (https://webblast.ipk-gatersleben.de/misa/) (Beier et al., 2017), employing the parameters “1-10 2-5 3-4 4-3 5-3 6-3”. Subsequently, tandem repeats were detected using the TRF tool (https://tandem.bu.edu/trf/trf.html) (Benson, 1999) with the parameters “2 7 7 80 10 50 500”. Interspersed repeats were discerned through the REPuter web server (https://bibiserv.cebitec.uni-bielefeld.de/reputer) (Kurtz et al., 2001), specifying the parameters “-c -f -p -r -l 30 -h 3 -best 50”. Repetitive sequences were visualized using Origin software.

### 2.4 Codon usage analysis

The coding sequences (CDS) of the mitogenome were extracted using Geneious R9.0.2 software (Kearse et al., 2012). The codon preferences for protein-coding genes (PCGs) were analyzed with CodonW software (http://codonw.sourceforge.net/), and relative synonymous codon usage (RSCU) values were calculated. An RSCU value greater than 1 indicates a codon is used more frequently than expected under equal usage assumptions. Stacked bar charts were generated using the ggplot2 package in R.

### 2.5 RNA editing site prediction

RNA editing, a post-transcriptional modification, is ubiquitously observed in eukaryotes, including plants. It entails alterations such as the addition, deletion, or conversion of bases within the coding region of transcripts (Jobson and Qiu, 2008; Wang et al., 2024b). To predict RNA editing sites in *Q. litseoides*, we employed the Deepred-Mt software (Edera et al., 2021). This tool utilizes a convolutional neural network (CNN) model and shows higher accuracy than previous methods (Edera et al., 2018). We chose results with probability values greater than 0.9.

### 2.6 Gene transfer between organelles

Previous research suggests the possibility of genetic material transfer between cellular compartments or organelles (Huang et al., 2003; Wang et al., 2024a). This study investigated gene transfer between organelles in *Q. litseoides*. We retrieved its chloroplast genome (NCBI: ON598394) and performed BLASTn analysis (v2.9.0) with the following parameters: an E-value of ≤ 1e−5, match rate of ≥ 70%, and alignment length of ≥ 40 bp. The results of the BLAST pairwise sequence alignment were subsequently visualized using the circlize package in R.

### 2.7 Sequence collinearity analysis

To explore the conserved mitochondrial regions among closely related species, including *Castanea henryi*, *C. mollissima*, *Castanopsis carlesii*, *Fagus sylvatica*, *Lithocarpus litseifolius*, *Quercus acutissima*, *Q. cerris*, *Q. ilex*, *Q. petraea*, *Q. robur*, and *Q. variabilis*, we performed pairwise alignments utilizing BLASTn (Chen et al., 2015). We retained only matches that exceeded 500 bp in order to evaluate the synteny patterns of the mitogenomes under investigation. Subsequently, we visualized the synteny of multiple genomes using NgenomeSyn v1.41 (He et al., 2023).

### 2.8 Phylogenetic analyses

To investigate the evolutionary placement of *Q. litseoides* within *Quercus*, we performed phylogenetic analyses based on both mitochondrial and chloroplast genomes. Eight *Quercus* species with available complete mitogenomes were included: *Quercus acutissima*, *Q. cerris*, *Q. chenii*, *Q. ilex*, *Q. litseoides*, *Q. petraea*, *Q. robur*, and *Q. variabilis*. *Fagus sylvatica* was selected as an outgroup to root the phylogenetic trees. Accession numbers and genome sources for the nine species were provided in **Supplementary Table S1**.

For mitochondrial phylogeny reconstruction, 29 shared mitochondrial PCGs were identified using the PhyloSuite v1.2.3 software and extracted for analysis (Xiang et al., 2023; Zhang et al., 2020). For chloroplast phylogeny reconstruction, the complete chloroplast genomes were used. The multiple sequence alignments were executed via the MAFFT v7.524 software with default parameters (Katoh and Standley, 2013). Phylogenetic trees were reconstructed using the Maximum Likelihood (ML) method implemented in the IQ-TREE v2.1.3 software, with automatic model selection (-m TEST) and 1,000 ultrafast bootstrap replicates (-bb 1000) to assess nodal support (Nguyen et al., 2015). The phylogenetic analysis results were ultimately visualized through FigTree v1.4.4 software.

## 3 Results

### 3.1 *Quercus litseoides* mitogenome features

**Figure 1:**
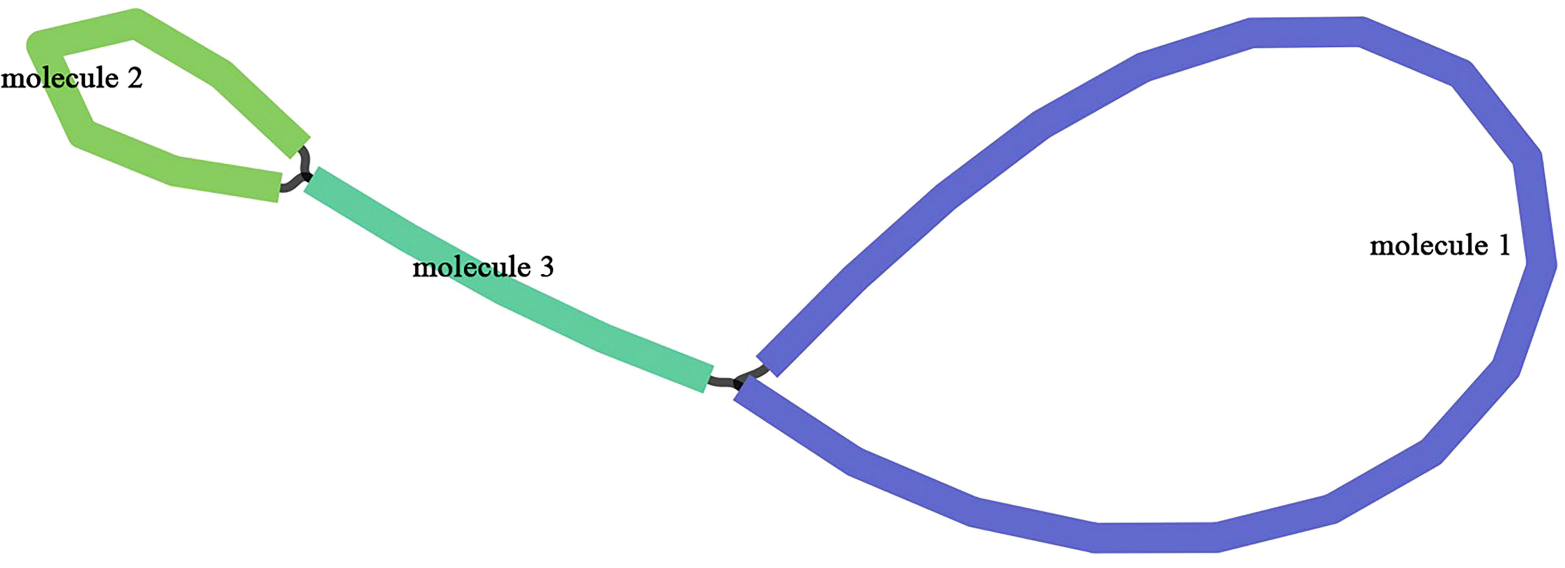
Branching topology of the *Q. litseoides* mitogenome.

**Figure 2:**
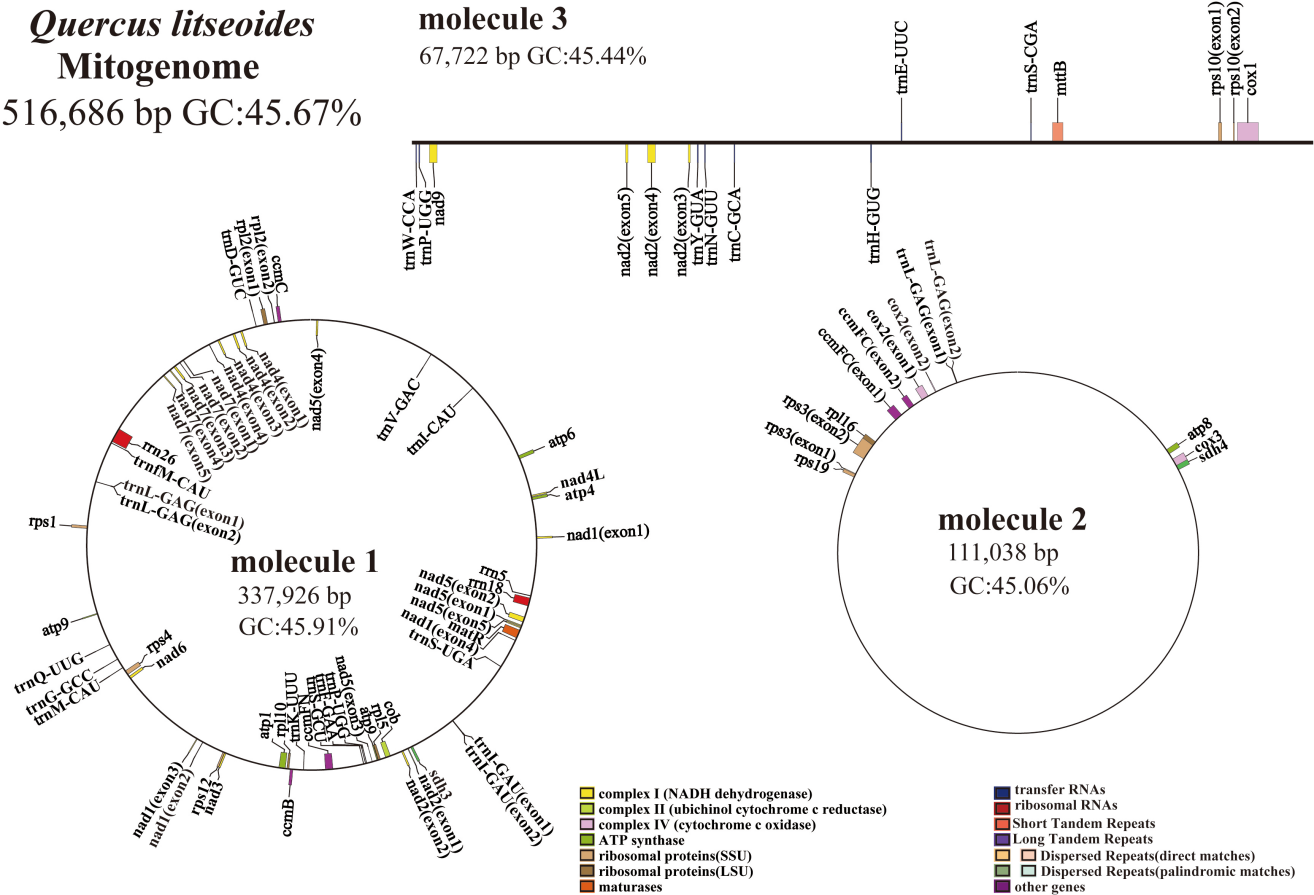
The mitogenome map of *Q. litseoides* is organized such that genes located on the outer circle and outside of it are transcribed in a clockwise direction, whereas those found on the inner circle and within it are transcribed in a counterclockwise direction. Different colors are used to identify genes associated with distinct functions.

The mitogenome of *Q. litseoides* displayed a multipartite architecture, comprising three discrete molecules. The assembly revealed two circular and one linear mitochondrial DNA molecules, totaling 516,686 bp with 45.67% GC content (**Figure 1**). Specifically, the circular molecules 1 and 2, along with the linear molecule 3, have lengths of 337,926 bp, 111,038 bp, and 67,722 bp respectively, with corresponding GC contents of 45.91%, 45.06%, and 45.44% (**Figure 2**). Coverage depth analysis revealed that all three molecular structures exhibited continuous and uniform coverage curves, with no low-depth or ambiguous regions detected at junctions or repeat regions (**Supplementary Figure S1**).

In the mitogenome of *Q. litseoides*, a total of 38 PCGs are annotated, comprising 25 mitochondrial core genes and 13 non-core genes, along with 23 transfer RNA genes (tRNAs) (*trnL-GAG* and *trnP-UGG* being multi-copies) and three ribosomal RNA genes (rRNAs). The core genes comprised five ATP synthase genes (*atp1*, *atp4*, *atp6*, *atp8*, and *atp9*), nine NADH dehydrogenase genes (*nad1*, *nad2*, *nad3*, *nad4*, *nad4L*, *nad5*, *nad6*, *nad7*, and *nad9*), four ubiquinone cytochrome c reductase genes (*ccmB*, *ccmC*, *ccmFC*, and *ccmFN*), three cytochrome c oxidase genes (*cox1*, *cox2*, and *cox3*), one transmembrane protein gene (*mttB*), one maturase gene (*matR*), and one cytochrome c biogenesis gene (*cob*). Notably, *atp9* was duplicated (**Table 1**). The exon-intron organization suggested that *nad1*, *nad2*, and *nad7* each contained five exons (**Supplementary Figure S2**). While *nad7* showed *cis*-splicing, other genes exhibited trans-splicing (**Supplementary Figure S3**), indicating distinct splicing mechanisms in *Q. litseoides*.

**Table 1:** Gene composition in the *Q. litseoides* mitogenome.

Group of genes	Name of genes
ATP synthase	*atp1, atp4, atp6, atp8, atp9* (×2)
NADH dehydrogenase	***nad1*** ****, ***nad2*** ****, nad3, nad4***, nad4L, ***nad5*** ***, nad6, nad7****, nad9
Cytochrome c biogenesis	ccmB, ccmC, ccmFC*, ccmFN
Ubiquinol cytochrome c reductase	*cob*
Cytochrome c oxidase	cox1, cox2*, cox3
Maturases	*matR*
Transport membrane protein	*mttB*
Large subunit of ribosome	rpl10, rpl16, rpl2*, rpl5
Small subunit of ribosome	rps1, rps10*, rps12, rps19, rps3*, rps4
Succinate dehydrogenase	*sdh3* (×2)*, sdh4*
rRNA	*rrn18, rrn26, rrn5*
tRNA	trnI-GAU*, trnL-GAG (×2)*, trnS-CGA, trnC-CGA, trnD-GUC, trnE-UUC, trnF-GAA, trnG-GCC, trnH-GUG, trnK-UUU, trnM-CAU, trnfM-CAU, trnI-CAU, trnN-GUU, trnP-UGG (×2), trnQ-UUG, trnS-GCU, trnS-UGA, trnV-GAC, trnW-CCA, trnY-GUA

(×2): Duplicated genes, *Number of introns, **Bold**: *Trans*-splicing genes.

### 3.2 Mitogenome repeats analyses

**Figure 3:**
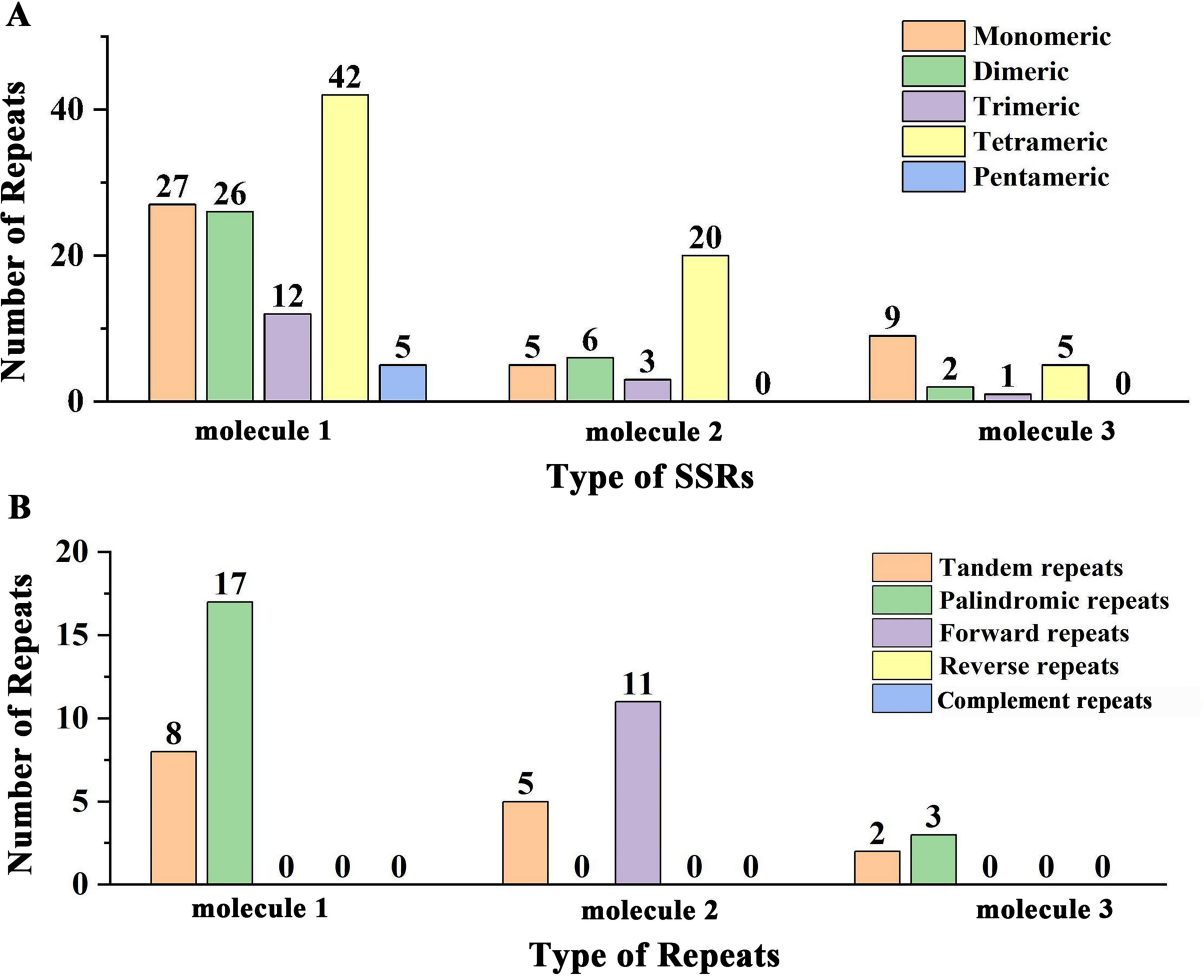
The repeat sequences of the mitogenome of *Q. litseoides*. **(A)** Total number of SSRs across various types. The x-axis signifies the types of the SSRs, while the y-axis denotes the number of SSRs. Each colored legend on the graph corresponds to a different SSR type: orange for monomers, green for dimers, purple for trimers, yellow for tetramers, and blue for pentamers. **(B)** Total number of repeats including tandem repeat sequences and interspersed repeat sequences. Here, the x-axis specifies the repeat types and the y-axis indicates their prevalence. The legend colors represent tandem (orange), palindromic (green), and forward repeats (purple).

In the mitogenome of *Q. litseoides*, we identified 112, 34, and 17 SSRs in molecule 1, molecule 2, and molecule 3, respectively (**Figure 3A**; **Supplementary Table S2**). Tetrameric repeats constituted the predominant repeat type in this mitogenome, with 67 identified instances, accounting for 41.10% of all SSRs. In addition, 41 monomeric repeats (25.15%) and 34 dimeric repeats (20.86%) were identified, although they were less abundant than tetrameric repeats. Notably, pentameric repeats occurred exclusively in circular molecule 1, while hexameric repeats were absent throughout the mitogenome. The composition analysis revealed that most SSRs consisted of adenine (A) and thymine (T) base pairs. Within the mitogenome of *Q. litseoides*, we detected 15 tandem repeats (**Figure 3B**; **Supplementary Table S3**). Among interspersed repeat sequences, only palindromic and forward repeats were identified, with no reverse or complement repeats detected. Palindromic repeats were found in molecule 1 and 3 (17 and 3 pairs, respectively), while forward repeats were restricted to molecule 2 (11 pairs) (**Figure 3B**). All interspersed repeats exceeded 30 bp in length (**Supplementary Table S4**).

### 3.3 Codon usage analysis

**Figure 4:**
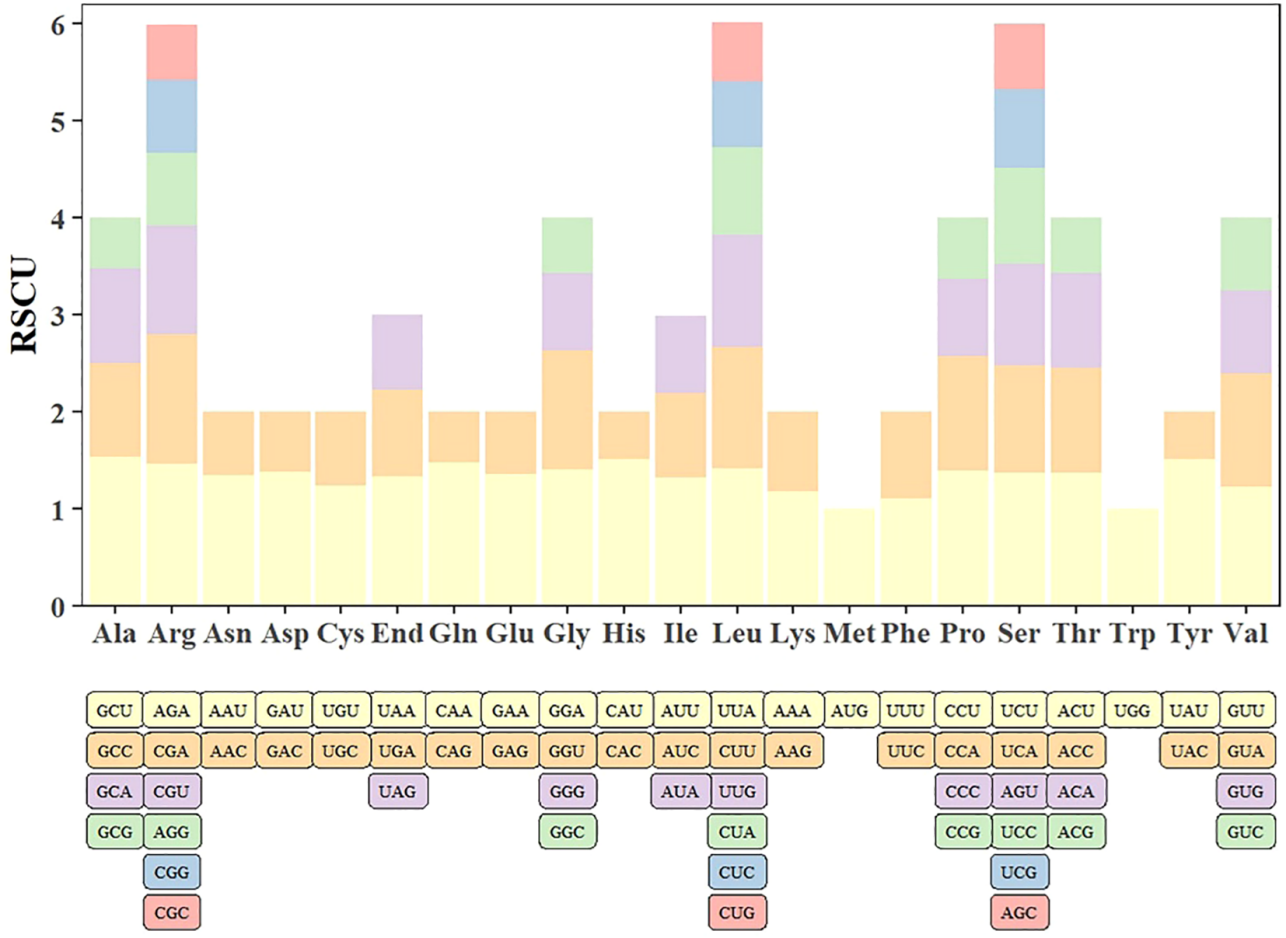
Codon preference analysis of the *Q. litseoides* mitogenome.

The codon usage bias was analyzed in 28 screened PCGs of the *Q. litseoides* mitogenome. **Supplementary Table S5** summarized the distribution of codon usage for each amino acid. The analysis revealed significant codon preference, with a total of 29 codons showing RSCU values > 1 (**Figure 4**; **Supplementary Table S5**). Among these, GCU (Ala), UAU (Tyr), and CAU (His) were the three most frequently used codons in *Q. litseoides*. We observed that most preferred codons ended with uracil (U), which may reflect mitochondrial tRNA abundance or mutational bias.

### 3.4 Prediction of RNA editing

**Figure 5:**
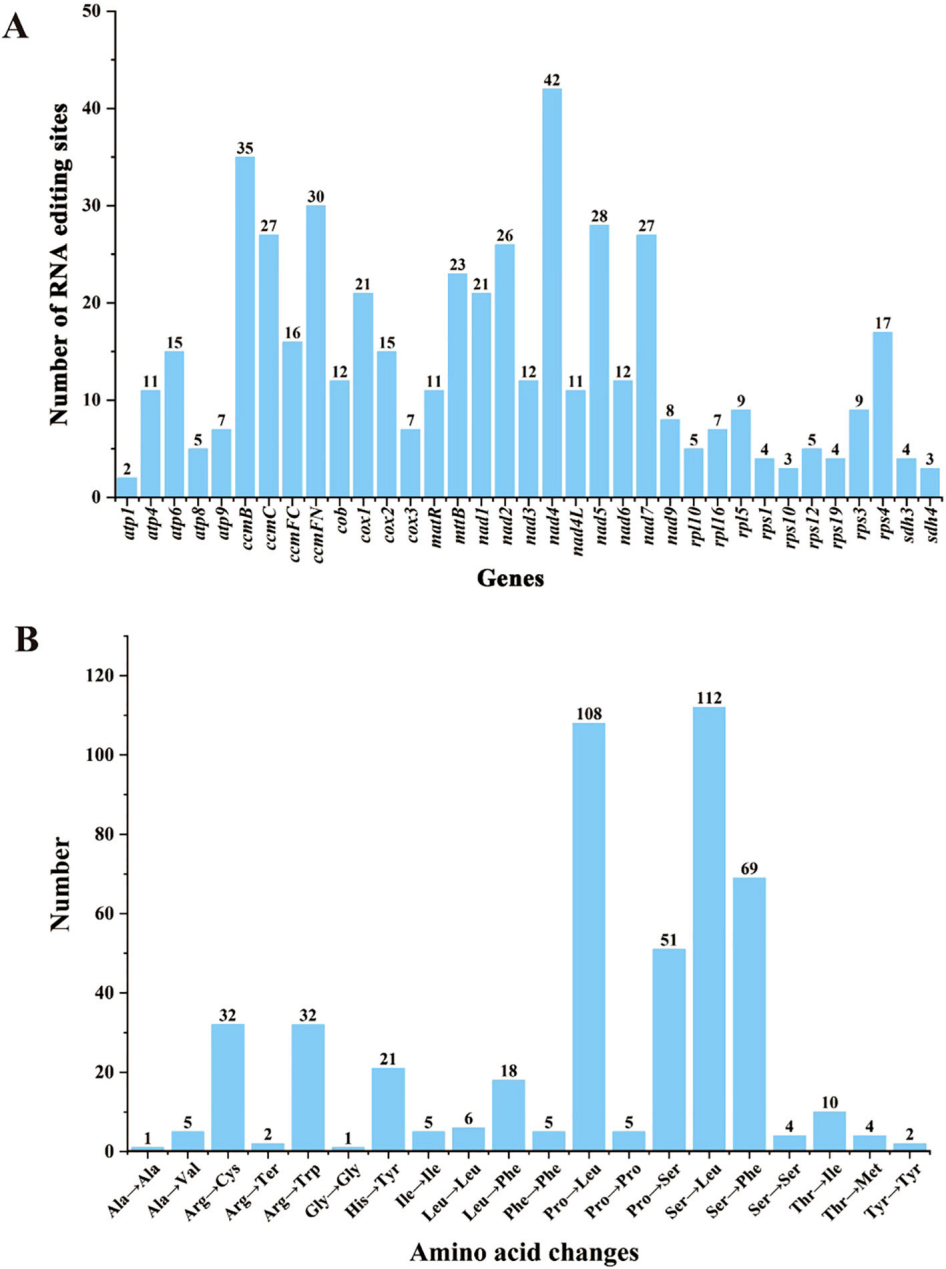
Characteristics of predicted RNA editing sites in 36 PCGs of the *Q. litseoides* mitogenome. **(A)** The number of predicted RNA editing sites in PCGs. **(B)** The number of amino acid changes caused by RNA editing sites.

Using the deep representation learning method (Deepred-Mt), we identified 494 C-to-U RNA editing sites across 36 PCGs in the *Q. litseoides* mitogenome (**Supplementary Table S6**). The distribution analysis showed that *nad4* contained the most editing sites (n=42), followed by *ccmB* (n=35), while *atp1* had only two editing sites (**Figure 5A**). Among these sites, 464 (93.93%) caused non-synonymous changes, while 30 (6.07%) remained synonymous. These non-synonymous editing events resulted in 12 distinct amino acid substitutions. The most frequent substitutions were serine (Ser) to leucine (Leu) (n=112) and proline (Pro) to leucine (Leu) (n=108) (**Figure 5B**), suggesting potential functional implications for mitogenome protein stability.

### 3.5 Gene transfer between organelles

**Figure 6:**
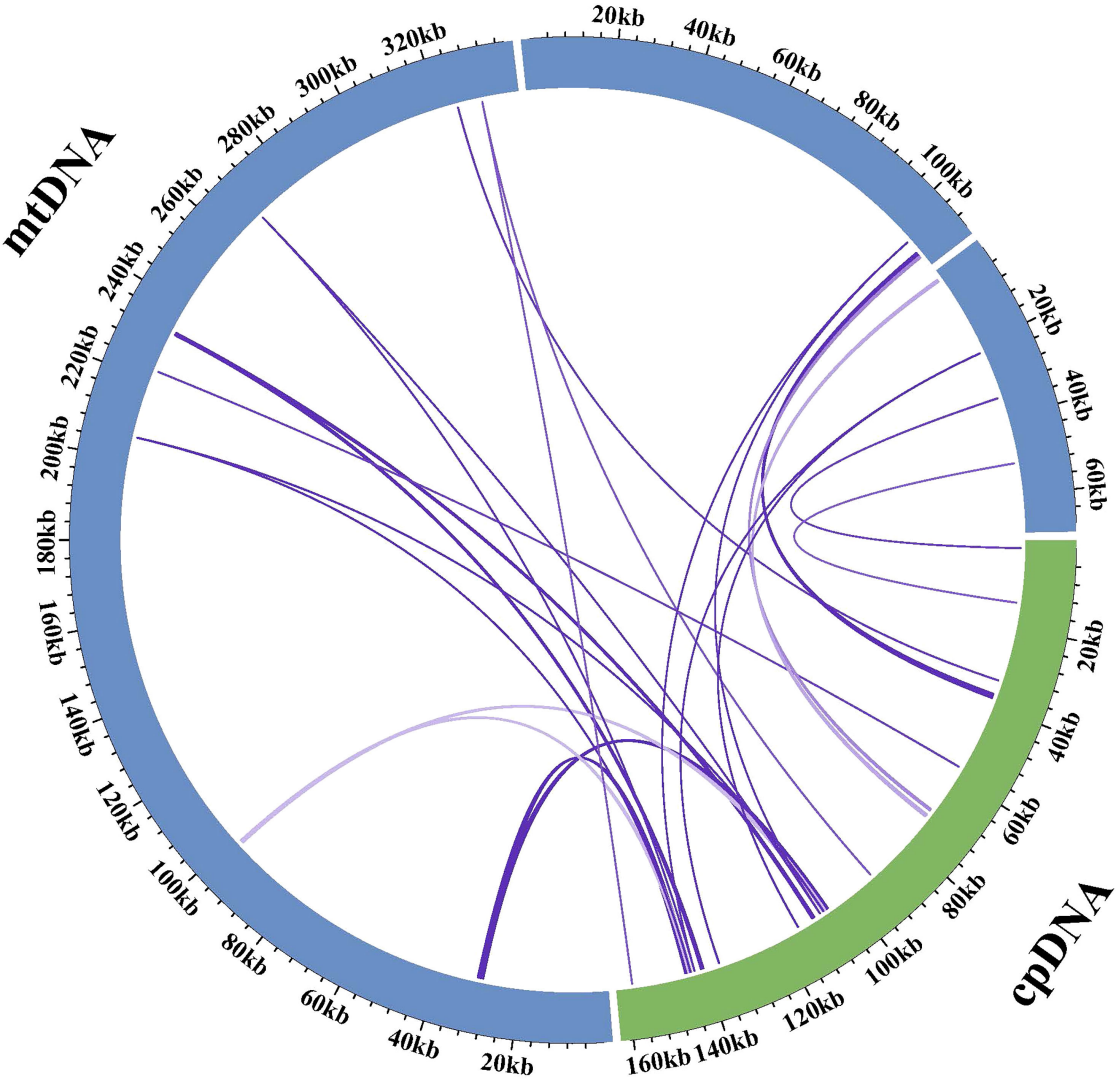
A schematic representation of gene transfer between organelles in *Q. litseoides*. The green and blue arcs symbolize the chloroplast and mitogenomes, respectively, while the purple lines connecting these arcs denote MTPTs.

In the mitogenome of *Q. litseoides*, we identified 15 homologous fragments shared with the chloroplast genome (excluding sequences aligned with chloroplast inverted repeat regions) (**Figure 6**; **Supplementary Table S7**). These chloroplast-derived sequences transferred to the mitogenome were designated as MTPTs. These identified homologous fragments ranged from 64 to 1,448 bp in length, totaling 6,183 bp (approximately 1.20% of the mitogenome). Annotation of these sequences identified two PCGs (*atpF* and *petG*), ten tRNA genes (*trnV-GAC*, *trnV-GAC*, *trnA-UGC*, *trnD-GUC*, *trnM-CAU*, *trnI-CAU*, *trnW-CCA*, *trnP-UGG*, *trnH-GUG*, and *trnN-GUU*), and one rRNA gene (*rrn16S*). These results demonstrated extensive gene transfer between organelles in *Q. litseoides*. The gene content analysis suggested that most transferred sequences were tRNA genes, indicating potential functional retention of these chloroplast-derived sequences in mitogenomes.

### 3.6 Synteny analysis

**Figure 7:**
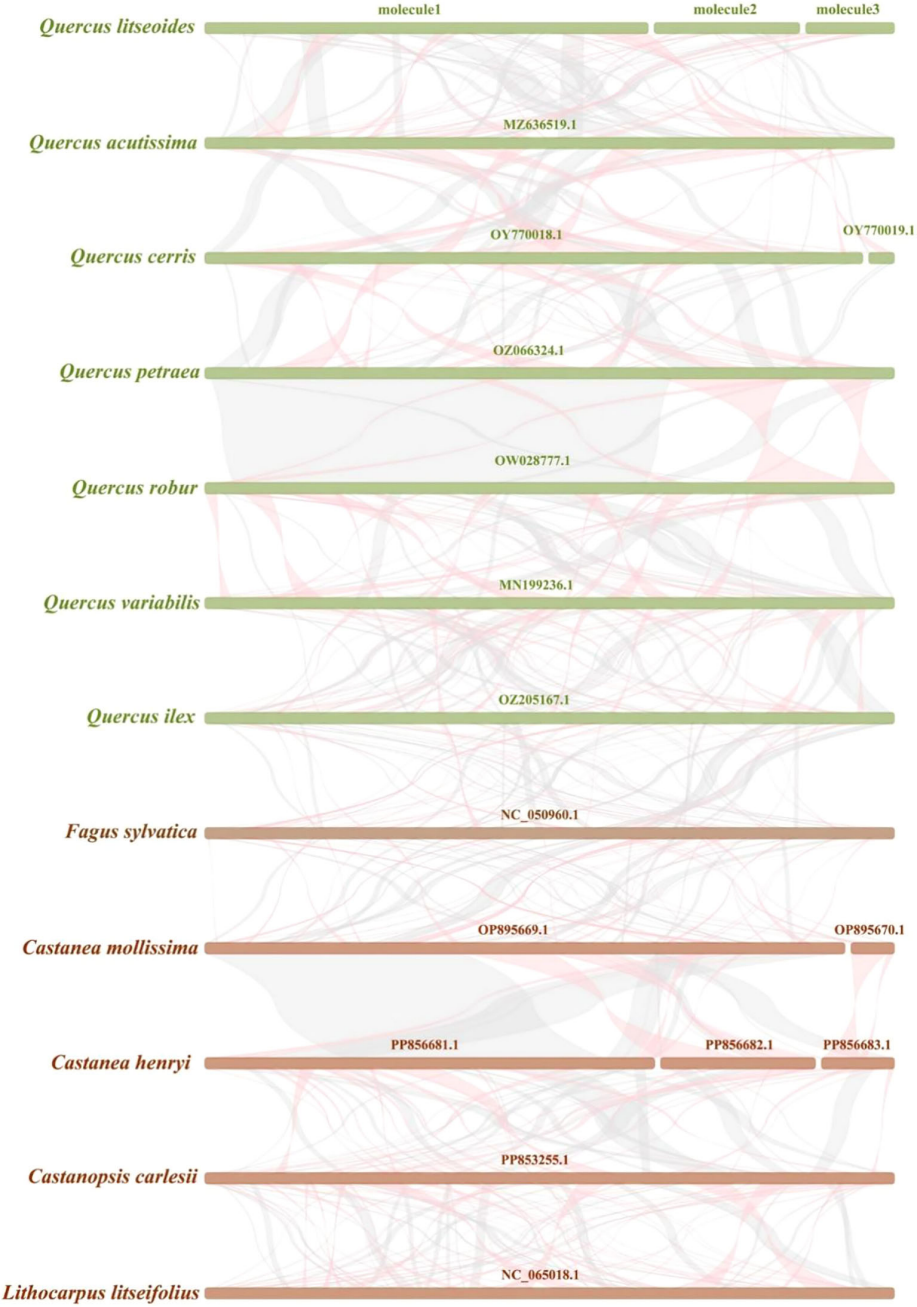
Synteny between *Q. litseoides* and closely related species. The red curved regions indicate inverted sequence regions, while the gray regions represent homologous sequence regions.

Homologous block analysis was conducted for 12 species of Fagaceae family. The multi-synteny plot clearly indicated several homologous collinear regions between *Q. litseoides* and its closely related species (**Figure 7**). For instance, the length of the longest matching fragment between *Q. litseoides* and *Q. acutissima* was 27,460 bp (**Supplementary Table S8**). However, these homologous fragments were relatively short, indicating limited mitogenome structural conservation among these species. Furthermore, the observed gaps suggested unique genomic features in *Q. litseoides*, as most sequences lacked homology with other species. These findings demonstrated extensive gene rearrangements in the *Q. litseoides* mitogenome compared to its relatives.

### 3.7 Phylogenetic analyses

**Figure 8:**
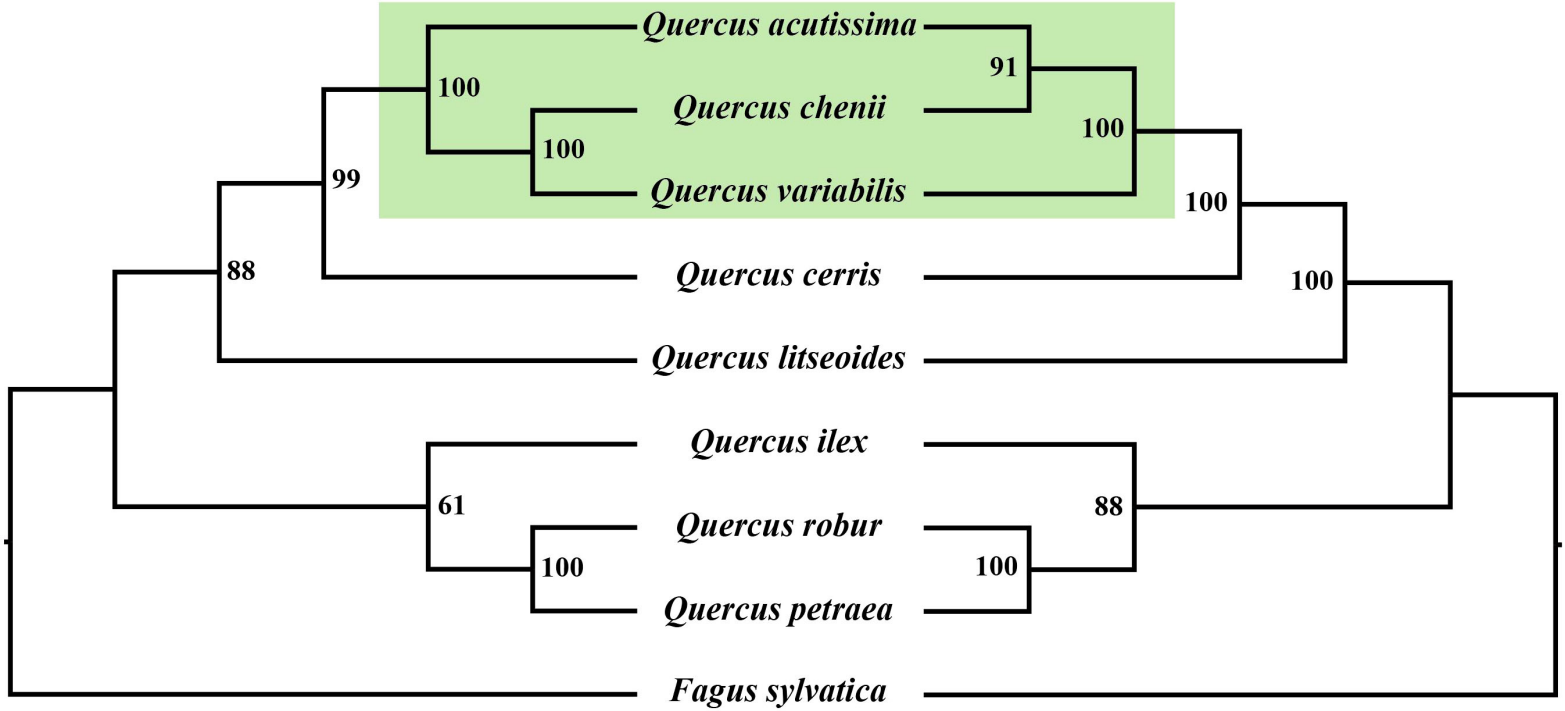
Maximum likelihood phylogenetic trees of eight *Quercus* species and *Fagus sylvatica*. **(A)** The tree is reconstructed based on mitogenomes. **(B)** The tree is reconstructed based on chloroplast genomes. The bootstrap support values (BS) are labeled at branch in the phylogenetic trees.

To explore the evolutionary relationships within *Quercus*, we reconstructed two phylogenetic trees using both mitochondrial and chloroplast genomes (**Figure 8**). The two phylogenetic trees exhibited similar topological structures, both with high bootstrap support values. Except for the differing placement of *Q. acutissima*, *Q. chenii*, and *Q. variabilis*, the remaining species showed consistent phylogenetic positions in both trees. *Quercus litseoides* was closely related to *Q. cerris* in both phylogenetic trees. Additionally, *Q. robur* and *Q. petraea* formed a strongly supported sister group (with 100% bootstrap support) in both trees, corroborating their classification within section *Quercus*. Overall, the phylogenetic analyses revealed both concordant and discordant evolutionary signals between mitochondrial and chloroplast genomes.

## 4 Discussion

### 4.1 Structural dynamics of the mitochondrial genomes

Mitochondria are essential organelles that function as cellular powerhouses and possess a highly complex genomic organization characterized by considerable sequence polymorphism and structural variation (Kozik et al., 2019). Although plant mitogenomes are typically depicted as circular molecules, recent studies have revealed that their actual structure *in vivo* is far more complex and dynamic, comprising a mixture of multimolecular forms, with linear DNA, circular and branched molecules (Gualberto et al., 2014; Kozik et al., 2019; Sloan et al., 2018). According to the most recent data accessible in the NCBI Genome Database (accessed: February 20, 2025), complete mitogenomes have been reported for 11 Fagaceae species, including *Quercus acutissima* (MZ636519.1) (Liu et al., 2022), *Q. variabilis* (MN199236.1, unverified) (Bi et al., 2019), *Q. cerris* (OY770018.1, unannotated), *Q. robur* (OW028777.1, unannotated), *Q. ilex* (OZ205167.1, unannotated), *Q. petraea* (OZ066324.1, unannotated), *Fagus sylvatica* (NC050960.1) (Mader et al., 2020), *Lithocarpus litseifolius* (NC065018.1) (Qiu et al., 2025), *Castanea henryi* (PP856681.1) (Tu et al., 2024), *C. mollissima* (OP895669.1) (Guo et al., 2023), and *Castanopsis carlesii* (PP853255.1) (Tu et al., 2024). Structural diversity within the Fagaceae mitogenomes is also well documented, such as the bi-circular structure in *C. mollissima* (Guo et al., 2023), the tripartite circular-linear organization in *Q. acutissima* (Liu et al., 2022) and the conventional single circle in *F. sylvatica* (Mader et al., 2020).

In this study, we reported the mitogenome of *Q. litseoides* from section *Cyclobalanopsis*, revealing a unique structure composed of two circular molecules and one linear molecule. This multipartite structure partially resembled that of *Q. acutissima* but differed in molecule number and composition, indicating lineage-specific structural diversification within *Quercus*. The mitogenome of *Q. litseoides* spanned 516,686 bp, which was intermediate in size between that of *Q. acutissima* (448,694 bp) and *L. litseifolius* (573,177 bp) (Liu et al., 2022; Qiu et al., 2025). Notably, it was the second longest among the currently known *Quercus* mitogenomes, following *Q. ilex*. The GC content serves as a crucial element in the evaluation of species (Liu et al., 2022). The GC content of this mitogenome was 45.67%, closely aligning with values observed in other Fagaceae species (such as *Q. acutissima* 45.72% and *Q. variabilis* 45.76%). This similarity suggests base composition conservation across this family, which may contribute to the maintenance of mitochondrial functional stability.

### 4.2 Structural stability and variation of the *Q. litseoides* mitogenome

Large repetitive sequences (>1 kb), commonly present in angiosperm mitogenomes as 2–3 copies, mediate homologous recombination to form characteristic multipartite structures (Cole et al., 2018; Odahara et al., 2021). Such recombination promotes genomic heteromorphism and structural diversity (Liu et al., 2022; Wynn and Christensen, 2019). In the *Q. litseoides* mitogenome, simple sequence repeat (SSR) analysis revealed that tetrameric repeats were the predominant type (41.10%), consistent with findings in other Fagaceae mitogenomes (Tu et al., 2024). However, the longest interspersed repeat (282 bp) was significantly shorter than those observed in *Q. acutissima* (10,578 bp) and *Fagus sylvatica* (918 bp), suggesting potentially constrained recombination activity (Liu et al., 2022; Mader et al., 2020). This low recombination trait may reflect an adaptive evolutionary strategy that helps maintain genome integrity and structural stability.

In parallel, codon usage analysis offered additional insights into the evolutionary constraints acting on the *Q. litseoides* mitogenome. Variation in codon usage frequency among eukaryotes is attributed to long-term evolutionary selection pressures (Tu et al., 2024). The RSCU value, indicating the ratio of observed codon usage frequency to the expected frequency under no bias, is crucial for evaluating species-specific codon preferences. Analyzing codon preferences is essential for elucidating the evolutionary dynamics of species (Sharp and Li, 1986). In the *Q. litseoides* mitogenome, a total of 29 codons exhibited RSCU values greater than 1, consistent with patterns observed in other Fagaceae species, suggesting a conserved codon usage bias within this family. This conservation likely contributes to translational efficiency and reflects selective constraints acting on mitochondrial gene expression.

### 4.3 Functional plasticity of the *Q. litseoides* mitogenome

RNA editing is a pivotal post-transcriptional regulatory mechanism in plant organelles (Edera et al., 2018), primarily involving cytosine-to-uracil (C-to-U) conversions that can alter amino acid sequences and expand protein diversity (Ma et al., 2022). In the *Q. litseoides* mitogenome, we detected a total of 494 RNA editing sites in 36 PCGs, all of which were C-to-U conversions. The most common amino acid transitions caused by these sites were serine-to-leucine (Ser-Leu) and proline-to-leucine (Pro-Leu), which was consistent with previous reports (Tu et al., 2024). Remarkably, RNA editing showed strong gene-specific variation, ranging from the highly edited *nad4* to the entirely unedited *rpl2*. These editing events are believed to contribute to translational diversity and may play a role in functional adaptation by modulating protein structure and function at the post-transcriptional level.

In addition to RNA editing, gene transfer between organelles also shapes the functional plasticity of plant mitogenomes. During the evolution of higher plants, mitogenomes have frequently incorporated fragments from plastid DNA, known as mitochondrial plastid DNA sequences (MTPTs) (Gui et al., 2016). Gene transfer between organelles is of evolutionary significance and has been widely observed as more mitogenomes and chloroplast genomes become available (Sloan and Wu, 2014). There is considerable variation in the length of the transferred fragments across various higher plant species (Gong et al., 2024). In this study, we identified 15 homologous fragments between the *Q. litseoides* organelles, representing 1.20% of the mitogenome and including 13 complete genes. Notably, the transfer of ten tRNA genes substantiates the frequent and functional exchange of genetic material between organelles in angiosperms (Bi et al., 2016). These transferred fragments not only provide raw materials for mitogenome remodeling but may also enhance functional diversification of the mitogenome.

Collectively, the observed RNA editing patterns and gene transfers between organelles highlight the functional flexibility of the *Q. litseoides* mitogenome. These features may reflect evolutionary adaptations that help maintain mitochondrial functionality under selective pressures, and they also offer valuable insights into the genetic mechanisms potentially related to the species’ endangered status.

### 4.4 Evolutionary insights of the mitochondrial genomes

Synteny analysis, which evaluates the sequence similarity of homologous sequences, is considered a powerful approach for investigating evolutionary relationships among species (Xu et al., 2023). In plant mitogenomes, the extent of synteny between two species can serve as an indicator of evolutionary distance and offer insights into their phylogenetic relationships. Moreover, rearrangements in plant mitogenomes play pivotal roles in enhancing genetic diversity, driving adaptive evolution, and regulating developmental and reproductive processes (Huang et al., 2025a). In this study, *Q. litseoides* exhibited extensive rearrangements compared to closely related species, reflecting a highly non-conserved and dynamic structural evolution. This phenomenon may be related to the structural plasticity of these mitogenomes. Despite these rearrangements, *Q. litseoides* retained relatively large syntenic blocks with *Q. acutissima*, *Q. cerris*, and *Q. ilex*, demonstrating conserved genomic regions and a shared evolutionary ancestry with these species. These results collectively underscore both the structural flexibility and the phylogenetic proximity of *Q. litseoides* within *Quercus*.

In this study, phylogenetic trees were reconstructed based on both mitochondrial and chloroplast genomes, and the results revealed highly consistent topological structures, highlighting the robustness of evolutionary relationships within *Quercus*. Notably, *Q. litseoides* consistently clustered with species from section *Cerris* in both trees, further supporting its phylogenetic placement and aligning with its traditional taxonomic classification within subgenus *Cerris* (Denk et al., 2017). Similarly, *Q. robur* and *Q. petraea*, both belonging to section *Quercus*, formed a strongly supported sister group (100% bootstrap support) in both trees, indicating their close evolutionary relationships and shared origin, consistent with previous morphological and molecular evidence (Denk et al., 2017; Hipp et al., 2020). Despite the overall consistency, slight discrepancies were observed in the phylogenetic positions of several species between the two organellar trees, particularly in *Q. acutissima*, *Q. chenii*, and *Q. variabilis*. These differences may reflect distinct evolutionary pressures acting on the mitochondrial and chloroplast genomes. In addition, factors such as introgression, historical hybridization, and incomplete lineage sorting are also considered major contributors to the observed phylogenetic incongruence between organelle genomes (Denk et al., 2017; Manos et al., 1999).

Taken together, these findings underscore the importance of integrating multiple organellar genomes to uncover subtle phylogenetic signals and lineage relationships within *Querus*. However, future studies incorporating nuclear genomic data will be essential to achieve a more comprehensive and robust understanding of *Quercus* evolution. Moreover, expanding the taxon sampling to include a broader range of *Quercus* species, especially those from underrepresented sections, will further refine phylogenetic resolution and enhance our understanding of diversification patterns within this ecologically and economically important genus.

## 5 Conclusion

This study successfully assembled the first complete mitogenome of *Q. litseoides* using PacBio HiFi long-read sequencing technology, filling the research gap in genomic data for *Quercus* section *Cyclobalanopsis*. The mitogenome spanned 516,686 bp with a GC content of 45.67% and exhibited a multipartite structure consisting of two circular molecules (circular molecule 1 and 2) and one linear molecule (linear molecule 3). Repeat analysis revealed that interspersed repeats were the primary contributors to mitogenome size variation within Fagaceae, while codon usage and RNA editing patterns were largely conserved across related species. Additionally, we detected 6,183 bp (1.20%) of mitochondrial plastid DNA sequences, indicating frequent gene transfer between organelles. Beyond expanding the genomic resources for Fagaceae, this mitogenome provides a valuable reference for future population-level studies. The identified repeats, RNA editing sites, and transferred genes offer candidate markers for assessing genetic diversity, population structure, and potential local adaptations in *Q. litseoides*. These genomic features can help identify conservation units, inform reintroduction efforts, and guide long-term genetic monitoring. Therefore, our findings not only enhance understanding of Fagaceae mitogenome evolution but also offer practical molecular tools to support conservation planning for this endangered and geographically restricted species.

## Data Availability

The raw sequencing data used in this study are publicly available at the China National Center for Bioinformation (CNCB) repository under accession number CRA027505 (https://ngdc.cncb.ac.cn/gsa/browse/CRA027505). The assembled and annotated mitogenome of Quercus litseoides has been deposited in the NCBI GenBank database under accession number PV558892 (https://www.ncbi.nlm.nih.gov/nuccore/PV558892).
