# Supplementary material for: Unraveling the mitochondrial genome of Quercus litseoides: a step towards conservation of an endangered species
Source: Front Plant Sci. 2025 Aug 19;16:1620373. doi: 10.3389/fpls.2025.1620373 (PMC12401974; doi:10.3389/fpls.2025.1620373)
Supplement: Supplementary file 2 [file DataSheet2.docx]

# Supplementary Figures

**
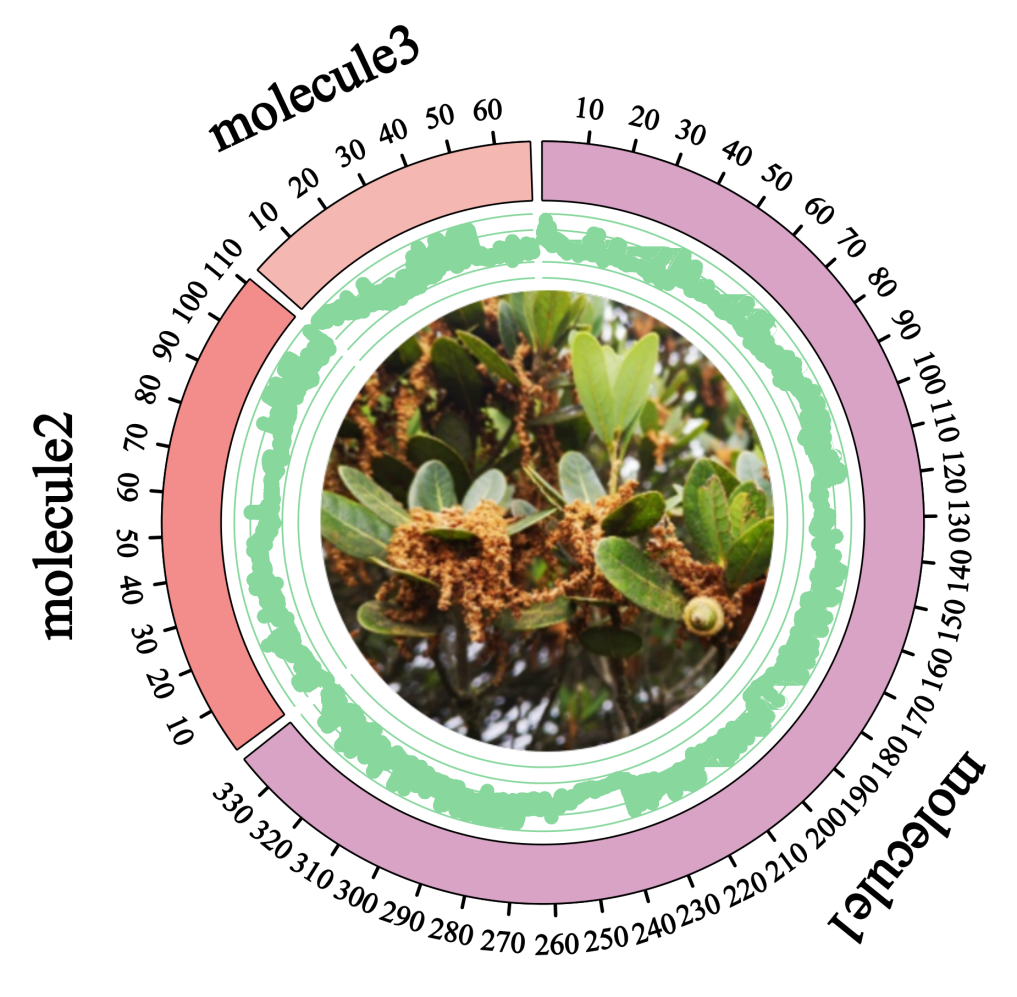
**

**Figure S1.** The coverage depth of the three assembled mitochondrial molecules in *Q. litseoides*.


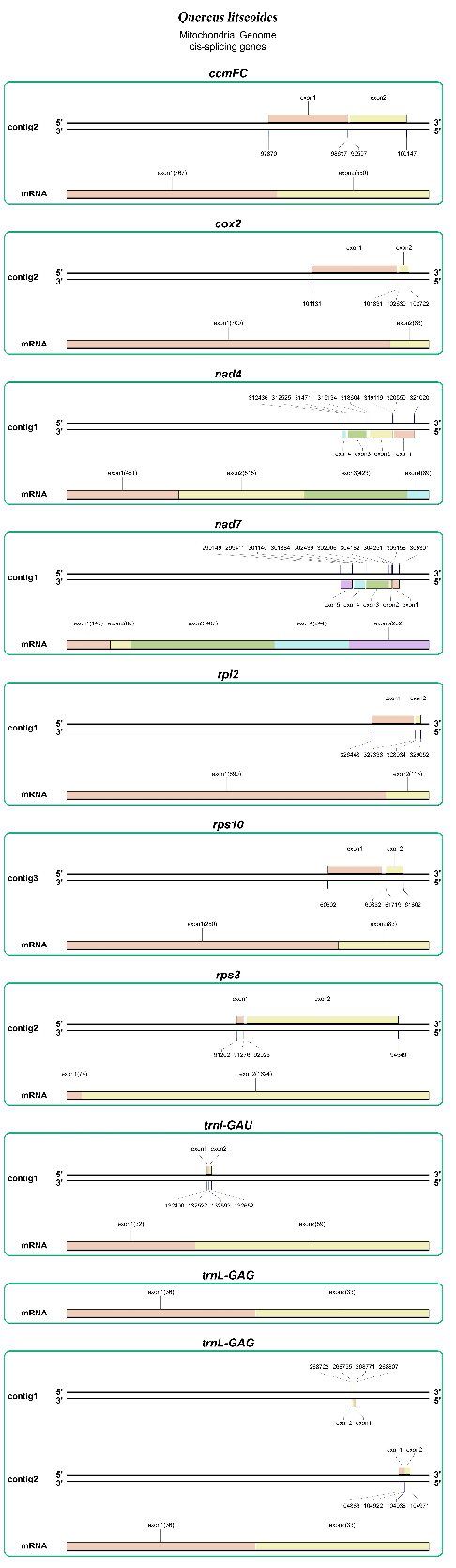


**Figure S2.** The *cis*-splicing gene of *Quercus litseoides*.


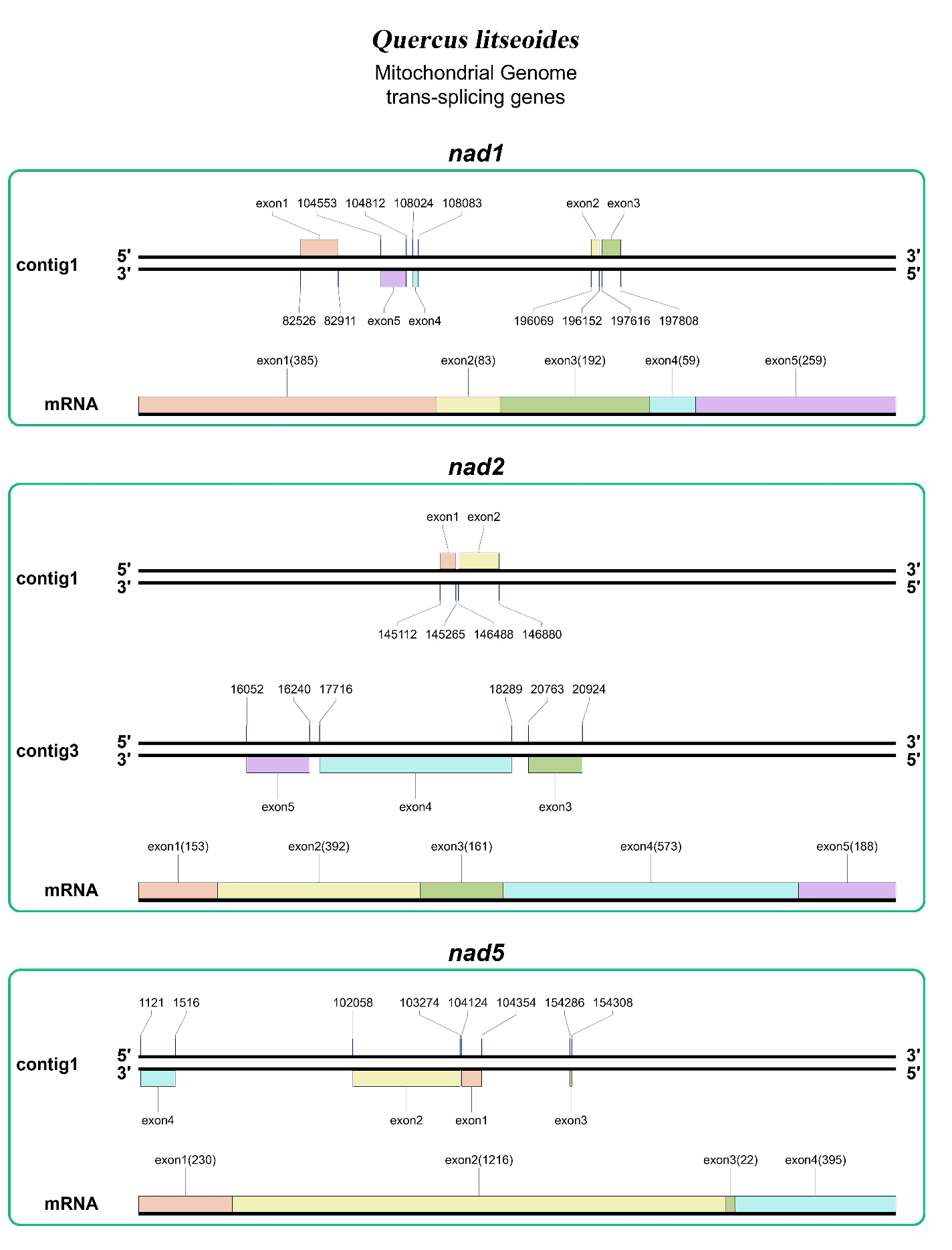


**Figure S3.** The *trans*-splicing gene of *Quercus litseoides*.
